# Peroral cholangioscopy-guided diagnosis and treatment of *Clonorchis sinensis* liver flukes

**DOI:** 10.1055/a-2333-9258

**Published:** 2024-06-07

**Authors:** Li-ying Tao, Hong-guang Wang, Qing-mei Guo, Shi-zhu Liu, Xiang Guo, Mu-yu Yang, Geng-jun Shi

**Affiliations:** 1 Department of Gastroenterology, Jilin People’s Hospital, Jilin, China

An 80-year-old man presented to our hospital with pain in the right upper quadrant of the abdomen that started 2 weeks prior to presentation. Laboratory analysis showed elevated C-reactive protein without leukocytosis. A transabdominal ultrasound showed dilated intrahepatic and extrahepatic bile ducts and gallbladder deposits. After obtaining informed consent, endoscopic ultrasonography (EUS) was pursued to explore the etiology for this biliary abnormality.

EUS revealed cholelithiasis, features suggestive of cholecystitis, and a dilated common bile duct (CBD) ( Fig. 1 **a, b** ). Endoscopic retrograde cholangiopancreatography with cholangioscopy was subsequently performed for further evaluation and management.

**Fig. 1:**
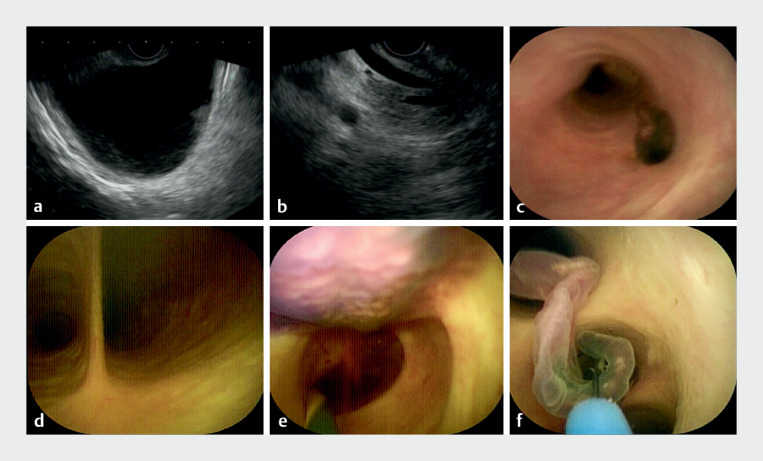
Imaging studies and endoscopy. **a, b** Endoscopic ultrasonography showed cholecystolithiasis, cholecystitis, and a dilated common bile duct (CBD). **c** The cholangioscope was introduced into the CBD and a *Clonorchis sinensis* body was visible. **d** The opening of the cystic duct was directly observed under cholangioscopy. **e** *C. sinensis* parasites were visible through direct cholangioscopy into the intrahepatic bile duct. **f** The *C. sinensis* body was removed using an elongated basket.

A cholangioscope was successfully introduced into the CBD and showed *Clonorchis sinensis* in the duct ( Fig. 1 **c** ). The opening of the cystic duct was clearly visualized under cholangioscopy ( Fig. 1 **d** ). *C. sinensis* parasites were also noted in the intrahepatic bile duct. We were unable to clear the biliary tree of *C. sinensis* despite copious irrigation and suction using the cholangioscope ( Fig. 1 **e** ). The *C. sinensis* body was then captured by an elongated basket and removed under direct cholangioscopic view through the duodenal papilla, in an atraumatic fashion ( Fig. 1 **f** , Video 1 **)** . Praziquantel was administered for 3 days after the procedure to treat *C. sinensis.*


Peroral cholangioscopy-guided diagnosis and treatment of *Clonorchis sinensis* liver flukes.
Video 1

Clonorchiasis is a common biliary parasitic disease 1 . The diagnosis of clonorchiasis is generally made by identifying the parasite eggs in fecal samples. However, our patient had tested negative for *C. sinensis* in stool. Studies have shown that cholangioscopy can improve the diagnostic rate of biliary diseases while reducing the use of X-rays 2 3 4 . In the present study, cholangioscopy facilitated the diagnosis and retrieval of biliary parasites.

Endoscopy_UCTN_Code_CCL_1AZ_2AI

## Notes

Endoscopy E-Videos https://eref.thieme.de/e-videos
*E-Videos* is an open access online section of the journal *Endoscopy* , reporting on interesting cases and new techniques in gastroenterological endoscopy. All papers include a high-quality video and are published with a Creative Commons CC-BY license. Endoscopy E-Videos qualify for HINARI discounts and waivers and eligibility is automatically checked during the submission process. We grant 100% waivers to articles whose corresponding authors are based in Group A countries and 50% waivers to those who are based in Group B countries as classified by Research4Life (see: https://www.research4life.org/access/eligibility/ ). This section has its own submission website at https://mc.manuscriptcentral.com/e-videos .
